# Spatially extended balanced networks without translationally invariant connectivity

**DOI:** 10.1186/s13408-020-00085-w

**Published:** 2020-05-13

**Authors:** Christopher Ebsch, Robert Rosenbaum

**Affiliations:** 1 Department of Applied and Computational Mathematics and Statistics, University of Notre Dame, Notre Dame, USA; 2 Interdisciplinary Center for Network Science and Applications, University of Notre Dame, Notre Dame, USA

**Keywords:** Balanced networks, Spiking neural network models, Excitatory-inhibitory balance, Mean-field theory

## Abstract

Networks of neurons in the cerebral cortex exhibit a balance between excitation (positive input current) and inhibition (negative input current). Balanced network theory provides a parsimonious mathematical model of this excitatory-inhibitory balance using randomly connected networks of model neurons in which balance is realized as a stable fixed point of network dynamics in the limit of large network size. Balanced network theory reproduces many salient features of cortical network dynamics such as asynchronous-irregular spiking activity. Early studies of balanced networks did not account for the spatial topology of cortical networks. Later works introduced spatial connectivity structure, but were restricted to networks with translationally invariant connectivity structure in which connection probability depends on distance alone and boundaries are assumed to be periodic. Spatial connectivity structure in cortical network does not always satisfy these assumptions. We use the mathematical theory of integral equations to extend the mean-field theory of balanced networks to account for more general dependence of connection probability on the spatial location of pre- and postsynaptic neurons. We compare our mathematical derivations to simulations of large networks of recurrently connected spiking neuron models.

## Introduction

Balanced networks [1, 2] offer a parsimonious computational and mathematical model of the asynchronous-irregular spiking activity and excitatory-inhibitory balance that are ubiquitous in cortical neuronal networks [3–10]. Balanced networks can produce asynchronous and irregular activity through chaotic or chaos-like spike timing dynamics [2, 11]. Mean-field analysis of balanced networks reveals a stable fixed point that naturally produces excitatory-inhibitory balance and weak pairwise spike train correlations without fine tuning of model parameters. In early studies, this mean-field analysis was performed in networks in which connection probabilities are homogeneous across the excitatory and inhibitory populations [1]. Later work extended the mean-field analysis to networks with multiple sub-populations and to spatially extended networks with distance-dependent connection probabilities [12–16], including models that combine physical and tuning space [17]. Previous work on spatially extended balanced networks assumed that connection probabilities depended only on the distance between neurons measured with periodic boundaries, rendering connection probabilities translationally invariant. This assumption allows the Fourier modes of network activity to decouple, so the mean-field equations can be easily solved in the Fourier domain. However, connectivity in cortical neuronal networks is not so simple. While the use of periodic boundaries is justified for modeling naturally periodic spaces like orientation tuning space, it is not necessarily realistic for models of physical space. Moreover, connection probabilities in cortical neuronal networks can depend on neuron location in more complicated ways than a pure distance dependence [18–20].

We use the mathematical theory of integral equations [21] to extend the mean-field theory of firing rates in balanced networks, permitting a more general dependence of connection probability on the spatial location of pre- and postsynaptic neurons. We derive conditions on the spatial structure connectivity and external input under which networks can maintain balance in the limit of large network size, derive the spatial profile of firing rates in this limit when balance is maintained, and derive a linear approximation to firing rates when balance is broken. We demonstrate our findings with simulations of large spiking networks under a simple spatial connectivity structure that violates the translational invariance of connection probabilities assumed by previous work.

## Model and background

We consider recurrent networks of *N* model neurons, $N_{e}=0.8N$ of which are excitatory and $N_{i}=0.2N$ inhibitory. The membrane potential of neuron *j* in population $a=e,i$ obeys the exponential integrate-and-fire dynamics

$$ C_{m}\frac{dV_{j}^{a}}{dt}=-g_{L}\bigl(V_{j}^{a}-E_{L} \bigr)+g_{L} D_{T} e^{(V_{j}^{a}-V_{T})/D_{T}}+I_{j}^{a}(t) $$

with the added condition that each time $V_{j}^{a}(t)$ exceeds $V_{th}$, it is reset to $V_{re}$, held for a refractory period of $\tau _{ref}$, and a spike is recorded at the threshold crossing time. The synaptic input current to neuron *j* in population *a* is given by

1 $$ I_{j}^{a}(t)=\sum _{b=e,i}\sum_{k=1}^{N_{b}} J_{jk}^{ab}\alpha _{b}\bigl(t-t_{n}^{a,k} \bigr)+ \sqrt{N} {F}^{a}_{j}, $$

where $t_{n}^{a,j}$ is the *n*th spike time of neuron *j* in population $a=e,i$ and $\alpha _{b}(t)=(1/\tau _{b})e^{-t/\tau _{b}}H(t)$ is a postsynaptic current waveform, and $H(t)$ is the Heaviside step function.

We consider a network on the compact domain $[0,1]$ with neuron $j=1,\ldots,N_{a}$ in population $a=e,i$ located at $x=j/N_{a}$. Connection strengths are defined by

2 $$ J_{jk}^{ab}= \textstyle\begin{cases} {j_{ab}}/{\sqrt{N}} & \text{with probability }p_{ab}(x,y), \\ 0 & \text{otherwise}, \end{cases} $$

where $x=j/N_{a}\in \varOmega $ is the location of (postsynaptic) neuron *j* in population *a* and $y=k/N_{b}\in \varOmega $ is the location of (presynaptic) neuron *k* in population *b*. We assume that $j_{ae}>0$ and $j_{ai}<0$ with all $j_{ab}$ constant with respect to *N*. The model and mean-field theory are easily extended to the case where connection strength and neuron density are spatially inhomogeneous (see below). The $1/\sqrt{N}$ scaling of synaptic weights is a defining feature of balanced networks which naturally captures excitatory-inhibitory balance and asynchronous-irregular spiking activity observed in cortical recordings [1, 2, 22]. Recent work in cultured cortical populations shows that similar scaling laws emerge naturally and produce network dynamics consistent with the balanced state [23]. Feedforward external input to the network is modeled by

$$ {F}_{j}^{a}={F}_{a}(x), $$

where $x\in \varOmega $ is the location of neuron *j* in population *a*. This models the input from $\mathcal{O}(N)$ neurons with synaptic weights that scale like $\mathcal{O}(1/\sqrt{N})$. Indeed, external input can be replaced by a population of generated spike trains without affecting the mean-field theory [17].

We consider a simple example in which $\varOmega =[0,1]$ and

3 $$ p_{ab}(x,y)=12\overline{p_{ab}}\bigl(\min (x,y)-xy\bigr), $$

where $\overline{p_{ab}}=\iint p_{ab}(x,y)\,dx\,dy$ is the network-averaged connection probability from *b* to *a*. Note that we use the convention that $p_{ab}$, $j_{ab}$, etc. refer to connection probabilities and strengths from presynaptic population *b* to postsynaptic population *a*. Note that, unlike spatial balanced networks considered in previous work [12, 17], $p_{ab}(x,y)$ is not translationally invariant and cannot be written in terms of $x-y$. Specifically, neurons near the center of the domain send and receive more connections than neurons near the edges.

**Figure 1:**
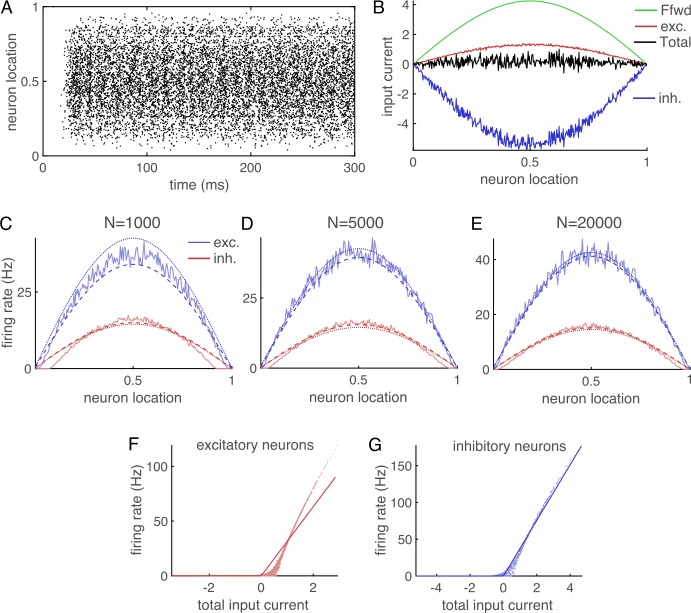
Example of a spatial balanced network without translational invariance and with simple sinusoidal external input. (**A**) Raster plot of excitatory neuron spikes from a simulated network with $N=5000$ neurons, recurrent connectivity given by Eqs. (2) and (3), and external input given by Eq. (4). (**B**) External input (green), mean recurrent excitatory input (red), mean recurrent inhibitory input (blue), and mean total input (black) to excitatory neurons as a function of neuron location for the same simulation as A. Currents were averaged over time (500 ms) and over the ten neurons nearest to each plotted location. Currents are computed with $C_{m}=1$ so are units $V/s$. (**C**)–(**E**) Firing rates of excitatory (red) and inhibitory (blue) neurons as a function of distance for $N=1000$, 5000, and $20\text{,}000$ respectively. Light solid curves are from simulations, dotted curves are from Eq. (14), and dashed curves from Eq. (15). Rates were averaged over all neurons in 200 evenly spaced bins and additionally averaged over $4\times 10^{5}/N$ simulations each with duration 10 s. (**F**) Firing rate versus mean total input current for all excitatory neurons with $N=5000$. Dots are from simulations and solid curve is the rectified linear fit used to derive the gain. (**G**) Same as F, but for inhibitory neurons

We first simulated this network with $N=5000$ neurons and

4 $$ {F}_{a}(x)=\overline{F}_{a} \sin (\pi x), $$

where $\overline{F}_{a}>0$. Our simulation produced asynchronous, irregular spiking activity (Fig. 1(A)) and excitatory-inhibitory balance (Fig. 1(B)) that are characteristic of the balanced network state [1, 2] and of cortical neuronal networks [3–10]. Note that the simulations are entirely deterministic once the random network connectivity and initial conditions are specified, so irregular spiking is driven by chaotic or chaos-like dynamics [2, 11], not noise. Firing rates were peaked near the center of the domain and decayed toward zero near the edge for various values of *N* (Fig. 1(C)–(E)). Firing rates that are peaked at the center of the spatial domain are not unexpected, given the structure of our connectivity kernel and input, and are common across many models of spatially extended networks [12, 24, 25]. We next derive a mean-field theory for computing firing rates in spatially extended balanced networks like this one.

Parameters for all figures are $C_{m}/g_{L}=15\text{ ms}$, $E_{L}=-72$ mV, $V_{T}=-60$ mV, $V_{th}=-15$ mV, $V_{re}=-72$, $V_{lb}=-100$, $\tau _{ref}=1\text{ ms}$, $\Delta _{T}=1.5$ mV, $\tau _{e}=8\text{ ms}$, $\tau _{i}=4\text{ ms}$, $j_{ee}/C_{m}=25$ mV, $j_{ei}/C_{m}=-150$ mV, $j_{ie}/C_{m}=112.5$ mV, $j_{ii}/C_{m}=-250$ mV, $\overline{F}_{e}/C_{m}=0.06$ V/s, $\overline{F}_{i}=0.05$ V/s, and $\overline{p}_{ee}=\overline{p}_{ei}=\overline{p}_{ie}=\overline{p}_{ii}=0.05$. Matlab and C code for running all simulations and generating all figures is publicly available at https://github.com/RobertRosenbaum/SpatialBalNetNonTransInvar/.

## Mean field theory of balance in spatially extended networks without translational invariance

The mean-field theory is developed by considering the large *N* limit and defining the mean-field firing rates

$$ {\boldsymbol{r}}(x)= \begin{bmatrix} r_{e}(x) \\ r_{i}(x)\end{bmatrix}, $$

where $r_{a}(x)$ is the expected value of the firing rates of a neuron in population *a* at location $x\in \varOmega $. The mean total input ${\boldsymbol{I}}(x)=[I_{e}(x) I_{i}(x)]^{T}$ and the feedforward external input ${\boldsymbol{{F}}}(x)=[{F}_{e}(x) {F}_{i}(x)]^{T}$ are defined analogously. We assume that ${F}_{a}\in L^{2}(\varOmega )$.

The following equation gives a heuristic approximation to neurons’ input as a function of firing rates in the network [12, 17]:

5 $$ {\boldsymbol{I}}=\sqrt{N} [\mathcal{W} {\boldsymbol{r}}+{\boldsymbol{{F}}} ]. $$

Here, $\mathcal{W}$ is an integral operator defined by

6 $$ \mathcal{W} {\boldsymbol{r}}= \begin{bmatrix} \mathcal{W}_{ee}r_{e}+\mathcal{W}_{ei}r_{i} \\ \mathcal{W}_{ie}r_{e}+\mathcal{W}_{ii}r_{i}\end{bmatrix}, $$

where

7 $$ [\mathcal{W}_{ab}r_{b}](x)= \int _{\varOmega }w_{ab}(x,y)r_{b}(y)\,dy, $$

$w_{ab}(x,y)=p_{ab}(x,y)j_{ab}q_{b}\sim \mathcal{O}(1)$ is the mean-field connectivity kernel with $q_{e}=N_{e}/N=0.8$, and $q_{i}=N_{i}/N=0.2$. We assume that $w_{ab}\in L^{2}(\varOmega ^{2})$ so that $\mathcal{W}_{ab}$ and $\mathcal{W}$ are Hilbert–Schmidt integral operators and are, therefore, compact [21]. We further assume that $w_{ab}(x,y)=w_{ab}(y,x)$ so that $\mathcal{W}_{ab}$ is a Hermitian integral operator (see Discussion for comments on relaxing some of these assumptions).

Note that $\mathcal{W}$ and ***F*** do not depend on *N*, but ***r*** and ***I*** do depend on *N*. In the balanced network theory below, we derive expressions for ***r*** that must be satisfied for $\|{\boldsymbol{r}}\|$ and $\|{\boldsymbol{I}}\|$ to be finite in the $N\to \infty $ limit (where $\|\cdot \|$ is the $L^{2}$ norm).

Equation (5) quantifies how firing rates are mapped to mean synaptic input in the network. A closed form approximation in Eq. (5) is possible in this case because the mapping is linear. However, the mapping from synaptic input to firing rates is necessarily nonlinear, depends on the details of the neuron model, can depend on higher moments of the input currents, and is generally not amenable to a closed-form mathematical approximation for the spiking network model considered here. Some studies use a diffusion approximation and Fokker–Planck formalism to account for the dependence of neurons’ firing rates on the first two moments of their input currents [26–28]. This approach can yield accurate approximations in practice, but makes the assumption that synaptic input is accurately approximated by Gaussian white noise, which may be inaccurate in some settings.

The mean-field theory of balanced networks offers an alternative approach to analyzing firing rates in which the mapping from synaptic input statistics to rates does not need to be known. This theory is developed and applied by analyzing Eq. (5) and the integral equations implied by it, which serves as a heuristic approximation to our spiking network model. We then compare the results of our analysis to simulations of the spiking network model. For balanced network theory, we do not need to specify the exact mapping from ***I*** to ***r***. Instead, the only necessary condition for the analysis of balanced networks is that the mapping does not converge to zero or diverge with *N*, more specifically that

8 $$ {\boldsymbol{r}}, {\boldsymbol{I}}\sim \mathcal{O}(1) $$

as $N\to \infty $, where all orders of magnitude expressions (expressions of the form $U\sim \mathcal{O}(F(N))$) should be interpreted in the $N\to \infty $ limit under the $L^{2}$ norm, so the expression above means $\lim_{N\to \infty }\|{\boldsymbol{r}}\|,\|{\boldsymbol{I}}\|<\infty $, where $\|\cdot \|$ is the $L^{2}$ norm on *Ω*. From Eq. (5), one can see that satisfying Eq. (8) implies that the network produces large $\mathcal{O}(\sqrt{N})$, excitatory and inhibitory synaptic currents that cancel or “balance” each other to produce $\mathcal{O}(1)$ total synaptic input. Hence, networks that satisfy Eq. (8) condition are said to be “balanced networks” or to operate in a “balanced state.”

Remarkably, this condition alone is enough to derive a linear, closed form expression for firing rates in the $N\to \infty $ limit even if the mapping from ***I*** to ***r*** is unknown. To see this, note that Eqs. (5) and (8) can only be realized when firing rates satisfy

9 $$ \mathcal{W} {\boldsymbol{r}}+{\boldsymbol{{F}}}=0 $$

in the $N\to \infty $ limit. In other words, if ${\boldsymbol{r}}_{\infty }=\lim_{N\to \infty } {\boldsymbol{r}}$ exists and is finite, then it must satisfy $\mathcal{W}{\boldsymbol{r}}_{\infty }+{\boldsymbol{{F}}}=0$. Note that $\mathcal{W}$ and ***F*** do not depend on *N*. Hence, firing rates in the $N\to \infty $ limit in balanced networks are determined by a linear integral equation of the first kind [21] despite the nonlinearity of the mapping from ***I*** to ***r***.

To better understand how this works, consider a commonly used integro-differential equation model for firing rates [24, 25] $\tau \dot{{\boldsymbol{r}}}=-{\boldsymbol{r}}+g({\boldsymbol{I}})$, where *g* is a monotonically increasing function. Any fixed point of this system satisfies $\mathcal{W}{\boldsymbol{r}}+{\boldsymbol{F}}=g^{-1}({\boldsymbol{r}})/\sqrt{N}$. As long as $g^{-1}({\boldsymbol{r}})/\sqrt{N}\to 0$ as $N\to \infty $ at the fixed point, any fixed point approaches the solution to Eq. (9) as $N\to \infty $.

Since Eq. (9) is a Fredholm integral equation of the first kind, it does not generically admit a solution. More specifically, for any integral operator $\mathcal{W}$, there necessarily exist external input profiles ${\boldsymbol{{F}}}(x)$, for which there is no solution ${\boldsymbol{r}}(x)$ to Eq. (9). When this occurs, the network cannot satisfy Eq. (8). Therefore, any spatially extended network can be imbalanced by some external input profiles. Moreover, solutions to Eq. (9) must be nonnegative and stable for the balanced state to be realized (see Discussion).

When Eq. (9) does not admit a solution, a linear approximation provides an approximation to firing rates at finite *N* [12, 17]. This approximation is obtained by making a rectified linear approximation to the mapping from mean input to mean firing rates $r_{a}(x)=g_{a}[I_{a}(x)]_{+}$ for $a=e,i$, where $g_{a}>0$ is the neurons’ gain and $[\cdot ]_{+}$ denotes the positive part. For spiking network models, $g_{a}$ can be approximated heuristically [12] or by fitting simulation results to a rectified linear function [17]. In the examples considered below, we use the latter approach. When firing rates are positive, this gives the following integral equation for firing rates:

10 $$ \mathcal{W} {\boldsymbol{r}}+{\boldsymbol{{F}}}=\epsilon D{\boldsymbol{r}}, $$

where $\epsilon =1/\sqrt{N}$ is a small, positive number and

$$ D= \begin{bmatrix} 1/g_{e}& 0 \\ 0 & 1/g_{i}\end{bmatrix}. $$

This equation is a finite *N* correction to Eq. (9). Since it is an integral equation of the second kind, it generically admits a unique solution for any ***F*** (unless *ϵ* is an eigenvalue of $\mathcal{W}$). Therefore, even when Eq. (9) does not admit a solution, Eq. (10) will generally admit a solution. However, the solution to Eq. (10) can diverge as $N\to \infty $ (*i.e.*, $\epsilon \to 0$), indicating networks for which Eq. (9) does not admit a solution.

A common approach to solving Fredholm integral equations like Eqs. (9) and (10) is to expand the equations using an orthonormal basis of eigenfunctions for the integral operator [21]. However, the integral operator is not guaranteed to have orthogonal eigenfunctions if it is not Hermitian. Because we assume $w_{ab}(x,y)=w_{ab}(y,x)$, the integral operators $\mathcal{W}_{ab}$ are Hermitian. However, the integral operator $\mathcal{W}$ that comprises our integral equations is not Hermitian because $w_{{ei}}\ne w_{{ie}}$. Hence, even though $\mathcal{W}_{ab}$ have orthogonal eigenfunctions, $\mathcal{W}$ does not. We extended the standard theory of integral equations to account for this case in which a non-Hermitian integral operator is composed of multiple Hermitian operators. Our extension is summarized by the following theorem. All convergences in the theorem and proof should be interpreted in an $L^{2}$ sense.

### Theorem 1

*Suppose that*$\mathcal{W}$*is defined as in Eqs*. (6) *and* (7) *where*$w_{ab}(x,y)=w_{ab}(y,x)$, $w_{ab}\in L^{2}(\varOmega ^{2})$, *and all four operators*$\mathcal{W}_{ab}$*share the same orthonormal basis of eigenfunctions*$\{\phi _{m}\in L^{2}(\varOmega )\}_{m}$*with associated eigenvalues*$\{\mu ^{ab}_{m}\}_{m}$. *When*$\epsilon \ne 0$*and*$\epsilon D-\widetilde{W}_{m}$*is nonsingular for allm*,

11 $$ {\boldsymbol{r}}(x)=\sum_{m} [\epsilon D- \widetilde{W}_{m} ]^{-1} \widetilde{{\boldsymbol{{F}}}}_{m} \phi _{m}(x) $$

*converges to a solution to Eq*. (10) *where*

$$ \widetilde{W}_{m}= \begin{bmatrix} \mu ^{{ee}}_{m} & \mu ^{{ei}}_{m} \\ \mu ^{{ie}}_{m} & \mu ^{{ii}}_{m}\end{bmatrix}, \qquad\widetilde{{ \boldsymbol{{F}}}}_{m}= \begin{bmatrix} \langle {F}_{e},\phi _{m}\rangle \\ \langle {F}_{i},\phi _{m}\rangle \end{bmatrix}, $$

*and*$\langle \cdot,\cdot \rangle $*is the*$L^{2}$*inner product onΩ*. *When the series in Eq*. (11) *converges at*$\epsilon =0$, *it converges to a solution to Eq*. (9).

### Proof

All convergences should be interpreted in the $L^{2}$ sense. Note that, since $w_{ab}(x,y)=w_{ab}(y,x)$ and $w_{ab}\in L^{2}$, $\mathcal{W}_{ab}$ is a self-adjoint Hilbert–Schmidt integral operator and is therefore a compact operator; therefore $\{\phi _{m}\}_{m}$ is a complete basis for $L^{2}(\varOmega )$ and $\mu _{m}^{ab}\in {\mathbb{R}}$ by the spectral theorem.

We first show that the series converges when $\epsilon \ne 0$. Note that $\mu _{m}^{ab}\to 0$ as $m\to \infty $ by the completeness of $\{\phi _{m}\}$ so that $\epsilon D-\widetilde{W}_{m}\to \epsilon D$ as $m\to \infty $ when $\epsilon \ne 0$. Therefore, the series in Eq. (11) converges only if the series $\sum_{m} [\epsilon D]^{-1} \widetilde{{\boldsymbol{F}}}_{m}\phi _{m}$ converges. The completeness of $\{\phi _{m}\}$ implies that $\sum_{m} \widetilde{{\boldsymbol{F}}}_{m}\phi _{m}$ converges since $\sum_{m} \langle {F}_{a},\phi _{m}\rangle \phi _{m}$ converges. We may conclude that the series in Eq. (11) converges.

We next show that the series in Eq. (11) converges to a solution to Eq. (10). Let ${\boldsymbol{r}}(x)=[r_{e}(x) r_{i}(x)]^{T}$ be defined by Eq. (11). Then

$$ \langle {\boldsymbol{r}},\phi _{m}\rangle \equiv \begin{bmatrix} \langle r_{e},\phi _{m}\rangle \\ \langle r_{i},\phi _{m}\rangle \end{bmatrix}= [ \epsilon D-\widetilde{W}_{m} ]^{-1} \widetilde{{ \boldsymbol{{F}}}}_{m}. $$

Multiplying both sides by $[\epsilon D-\widetilde{W}_{m} ]$ gives

$$ \epsilon D\langle {\boldsymbol{r}},\phi _{m}\rangle -\widetilde{W}_{m} \langle { \boldsymbol{r}},\phi _{m}\rangle =\widetilde{{\boldsymbol{{F}}}}_{m}, $$

which gives

$$ \langle \epsilon D {\boldsymbol{r}},\phi _{m}\rangle -\langle \mathcal{W} { \boldsymbol{r}},\phi _{m}\rangle = \langle {\boldsymbol{F}},\phi _{m}\rangle $$

for all *m*. Since $\phi _{m}$ is a complete basis, this implies that $\epsilon D {\boldsymbol{r}}- \mathcal{W} {\boldsymbol{r}}={\boldsymbol{F}}$ and therefore ***r*** satisfies Eq. (10).

Now assume that the series in Eq. (11) converges at $\epsilon =0$. Repeating the argument above with $\epsilon =0$ shows that it converges to a solution to Eq. (9) whenever it converges. □

Note that the convergence of Eq. (11) when $\epsilon >0$ is implied by the assumptions of the theorem, but the convergence when $\epsilon =0$ needs to be assumed separately. Hence, Eq. (10) admits solutions under relatively general assumptions, whereas the solvability of Eq. (9) is less general and requires that $\|\widetilde{{\boldsymbol{F}}}_{m}\|$ converges to zero faster than $\|\widetilde{W}_{m}\|$.

While the assumption of symmetric convolution kernels assures that $\mathcal{W}_{ab}$ have orthonormal bases of eigenfunctions with real eigenvalues, we additionally assumed that these eigenfunctions were the same for all four combinations of $a,b\in \{e,i\}$. This would be satisfied, for example, if $w_{ab}(x,y)=\overline{w}_{ab} k(x,y)$ for some $\overline{w}_{ab}\in {\mathbb{R}}$, but also applies to other settings. For example, if $w_{ab}(x,y)=w_{ab}(x-y)$ are periodic or “wrapped” convolution kernels as in previous work [12, 15, 17], then the Fourier basis functions provide an orthonormal basis of eigenfunctions even when the four convolution kernels $w_{ab}(x-y)$ are not multiples of each other. In this case, Eq. (11) is the Fourier series for ${\boldsymbol{r}}(x)$. In Discussion, we comment on how some of these assumptions could be weakened.

When $\mathcal{W}_{ab}$ do not share orthonormal bases of eigenfunctions, the analysis must be performed directly on the spectrum of $\mathcal{W}$ instead of decomposing the analysis into the spectra of each $\mathcal{W}_{ab}$. Specifically, if $\mathcal{W}$, treated as an integral operator on $L^{2}(\varOmega )\times L^{2}(\varOmega )$, has an orthonormal basis of eigenfunctions $\{\phi _{m}\}_{m}$ where $\phi _{m}\in L^{2}(\varOmega )\times L^{2}(\varOmega )$ with real eigenvalues $\{\mu _{m}\}$, then ${\boldsymbol{r}}=\sum_{m} [\epsilon D-\mu _{m}]^{-1}\langle {\boldsymbol{F}},\phi _{m} \rangle \phi _{m}$. However, note that $\mathcal{W}$ may not have an orthonormal basis of eigenfunctions with real eigenvalues even when kernels are spatially symmetric ($w_{ab}(x,y)=w_{ab}(y,x)$) because a Hermitian $\mathcal{W}$ would also require that $w_{ei}(x,y)=w_{ie}(y,x)$, which is generally not true for excitatory-inhibitory networks. Solving Fredholm equations with non-Hermitian integral operators is generally more difficult as diagonalization methods cannot be applied directly. Hence, our derivation of Eq. (11) extends standard diagonalization methods because it solves a Fredholm equation with a non-Hermitian kernel, albeit one with a special structure in which $\mathcal{W}$ is composed of multiple Hermitian kernels. This structure arises naturally in spatial neural network models with excitatory and inhibitory populations.

A corollary gives a simpler expression for the solution to Eq. (9) when the spatial shape of the connectivity kernels and external input are the same for excitatory and inhibitory neurons.

### Corollary 1

*Suppose*, *in addition to the assumptions of Theorem *1, *that*$w_{ab}(x,y)=\overline{w}_{ab} k(x,y)$*and*${F}_{a}(x)=\overline{{F}}_{a} {F}(x)$*for some*$\overline{w}_{ab},\overline{F}_{a}\in {\mathbb{R}}$. *Then a solution to Eq*. (9) *exists and is equal to*

12 $$ {\boldsymbol{r}}(x)=-\overline{W}^{-1}\overline{{\boldsymbol{{F}}}} \sum_{m} \frac{\widetilde{{F}}_{m}}{\mu _{m}}\phi _{m}(x) $$

*if the series converges*. *Here*,

$$ \overline{W}= \begin{bmatrix} \overline{w}_{{ee}} &\overline{w}_{{ei}} \\ \overline{w}_{{ie}} & \overline{w}_{{ii}}\end{bmatrix}, \qquad\overline{{\boldsymbol{{F}}}}= \begin{bmatrix} \overline{{F}}_{e} \\ \overline{{F}}_{i}\end{bmatrix}, $$

$\widetilde{{F}}_{m}=\langle {F},\phi _{m}\rangle $, *and*$\mu _{m}$*is the eigenvalue of the integral operator*$[\mathcal{K}r](x)=\int _{\varOmega }k(x,y)r(y)\,dy$*associated with eigenfunction*$\phi _{m}$.

Note that the first product in Eq. (12) (before the sum) is a vector (it has an excitatory and inhibitory component), but is not a function of *x*. On the other hand, the second term (the sum) is scalar, but depends on *x*. Hence, the solution is broken into its spatial and excitatory-inhibitory components.

If $\mathcal{K}$ has a nontrivial nullspace, then some of the eigenvalues $\mu _{m}$ will be zero. For Eq. (9) to be solvable in this case, $\widetilde{F}_{m}=\langle {F},\phi _{m}\rangle $ must be zero for all such *m* and the corresponding terms in the series in Eq. (12) should be interpreted as zero.

The same separation of terms cannot be applied to solving Eq. (10), *i.e.*, Eq. (11) cannot be simplified in the same way when $\epsilon \ne 0$. However, the evaluation of Eq. (11) when $\epsilon \ne 0$ is simplified to some degree by noting that $\widetilde{W}_{m}=\overline{W}\mu _{m}$ and $\widetilde{{\boldsymbol{F}}}_{m}=\overline{{\boldsymbol{F}}}\widetilde{F}_{m}$ under the assumptions of Corollary 1 and therefore

13 $$ {\boldsymbol{r}}(x)=\sum_{m} [\epsilon D- \overline{W} \mu _{m} ]^{-1} \overline{{\boldsymbol{{F}}}} \widetilde{{F}}_{m} \phi _{m}(x). $$

Since all the examples we consider satisfy the assumptions of Corollary 1, we hereafter focus on Eqs. (12) and (13) in place of the more general Eq. (11). The analysis of specific networks with specific external input profiles can proceed as follows: The convergence of the series in Eq. (12) should be checked first. If it does not converge, then the network cannot maintain balance as $N\to \infty $ and Eq. (13) must be used at finite *N* instead. Even when Eq. (12) does converge, Eq. (13) can be used as an alternative to Eq. (12) for approximating firing rates in spiking network simulations. We next compare Eqs. (12) and (13) to results from large spiking network simulations.

## Comparison of mean-field theory to spiking network simulations

We first consider the simulations discussed above and shown in Fig. 1. For this example, we can write $w_{ab}(x,y)=\overline{w}_{ab}k(x,y)$ where $\overline{w}_{ab}=12 \overline{p}_{ab}j_{ab}q_{b}$ and $k(x,y)=\min (x,y)-xy$, and we can write ${F}_{a}(x)=\overline{{F}}_{a} {F}(x)$ with ${F}(x)=\sin (\pi x)$. Therefore, we can apply Corollary 1. It is easily checked that the integral operator $[\mathcal{K}r](x)=\int _{\varOmega }k(x,y)r(y)\,dy$ has eigenvalues and orthonormal eigenfunctions

$$ \mu _{m}=\frac{1}{m^{2}\pi ^{2}} \quad\text{and}\quad \phi _{m}(x)= \sqrt{2} \sin (m\pi x) $$

for $m\in {\mathbb{N}}$. Since ${F}(x)=\sin (\pi x)$ is itself an eigenfunction, its expansion in the eigenfunction basis has only one nonzero term $\widetilde{{F}}_{1}=\langle {F},\phi _{a}\rangle =1/\sqrt{2}$ and $\widetilde{{F}}_{m}=0$ for $m\ne 1$. Therefore, the series in Eq. (12) has only one nonzero term and reduces to

14 $$ {\boldsymbol{r}}(x)=-\overline{W}^{-1}\overline{{\boldsymbol{{F}}}}\pi ^{2}\sin (\pi x), $$

which represents the $N\to \infty $ limit of firing rates in the balanced state. The finite *N* correction in Eq. (13) similarly has only one term and is given by

15 $$ {\boldsymbol{r}}(x)= \bigl[\pi ^{2}\epsilon D-\overline{W} \bigr]^{-1} \overline{{\boldsymbol{{F}}}}\pi ^{2} \sin (\pi x). $$

Comparing these expressions to firing rates computed from simulations shows that Eq. (15) provides a noticeably better approximation at $N=1000$ (Fig. 1(C); dashed is closer than dotted to solid), but both equations agree very well at larger values of *N* (Fig. 1(D), (E); dashed and dotted are close to solid), as expected.

Figure 1(F), (G) shows individual neurons’ firing rates versus their time-averaged input currents (dots) and compares them to the rectified linear fit used to estimate $g_{e}$ and $g_{i}$ (solid curves). Note that the rectified linear fit is not highly accurate, especially for excitatory neurons, because the true relationship between ***I*** and ***r*** is not a rectified linear function. However, this rough approximation is sufficient because $g_{e}$ and $g_{i}$ only affect Eq. (15) at order $\mathcal{O}(\epsilon )$.

We next consider a more interesting example in which the series in Eqs. (13) and (12) have infinitely many nonzero terms. In particular, consider the same recurrent network with external input

16 $$ {F}(x)=c\sin ^{4}(x)+(1-c) \sin (\pi x). $$

Since we have not changed the network, $\mu _{m}$ and $\phi _{m}(x)$ are the same as above and the only term that changes from above is

$$ \widetilde{{F}}_{m}=\frac{(1-c)}{\sqrt{2}}\delta _{m,1}+ \textstyle\begin{cases} \frac{48c\sqrt{2}}{(64m-20m^{3}+m^{5})\pi } & m\text{ odd}, \\ 0 & m\text{ even},\end{cases} $$

where *δ* is the Kronecker delta. It is clear then that the series in Eq. (12) converges since $\widetilde{{F}}_{m}/\mu _{m}\sim m^{-3}$ as $m\to \infty $. Indeed, one can simplify the series in Eq. (12) to obtain

17 $$\begin{aligned} {\boldsymbol{r}}(x)={}&{-}\overline{W}^{-1} \overline{{\boldsymbol{{F}}}} \bigl((1-c)\pi ^{2} \sin (\pi x) \\ &{}+2c\pi ^{2}\bigl[\cos (4\pi x)-\cos (2\pi x)\bigr] \bigr) \end{aligned}$$

as the firing rates in the $N\to \infty $ limit in the balanced state. This is only a valid firing rate solution when ${\boldsymbol{r}}(x)>0$ for all $x\in [0,1]$, which requires that *c* is sufficiently small. Specifically, *c* must be small enough that $(1-c)\sin (\pi x)+2c[\cos (4\pi x)-\cos (2\pi x)]\ge 0$ for all $x\in [0,1]$. For the finite *N* correction in Eq. (13), we were unable to obtain a closed form expression for the series, but it can easily be summed numerically using the equations for $\mu _{m}$ and $\widetilde{F}_{m}$ derived above.

**Figure 2:**
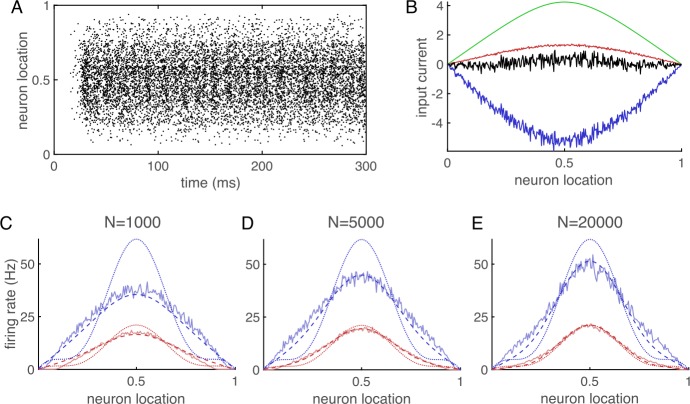
A second example of a spatially extended balanced network. (**A**)–(**E**) Same as Fig. 1 except external input is given by Eq. (16) with $c=0.15$, the dotted lines in (**C**)–(**E**) are given by Eq. (17), and the dashed lines are given by summing Eq. (13) numerically up to $m=20$

Network simulations show asynchronous-irregular spiking activity (Fig. 2(A)) and excitatory-inhibitory balance (Fig. 2(B)). Comparing our theoretical equations to firing rates from simulations shows that Eq. (13) is much more accurate than Eq. (17) even at larger values of *N*, but the convergence of the two equations to each other (and to results from simulations) is visible when comparing $N=5000$ to $N=20\text{,}000$ (Fig. 2(C)–(E)).

We finally consider the same recurrent network with external input

18 $$ {F}(x)=c\sin ^{2}(x)+(1-c) \sin (\pi x). $$

In this case,

$$ \widetilde{{F}}_{m}=\frac{(1-c)}{\sqrt{2}}\delta _{m,1}+ \textstyle\begin{cases} \frac{4c\sqrt{2}}{\pi (4m-m^{3})} & m\text{ odd}, \\ 0 & m\text{ even}.\end{cases} $$

Therefore, ${F}_{m}/\mu _{m} \sim m^{-1}$ as $m\to \infty $ and the series in Eq. (12) diverges. This implies that the network does not maintain excitatory-inhibitory balance as $N\to \infty $ because Eq. (9) does not admit a solution. Equation (10) still admits a solution for each *N*, given by Eq. (13), but this solution diverges as $N\to \infty $ ($\epsilon \to 0$).

**Figure 3:**
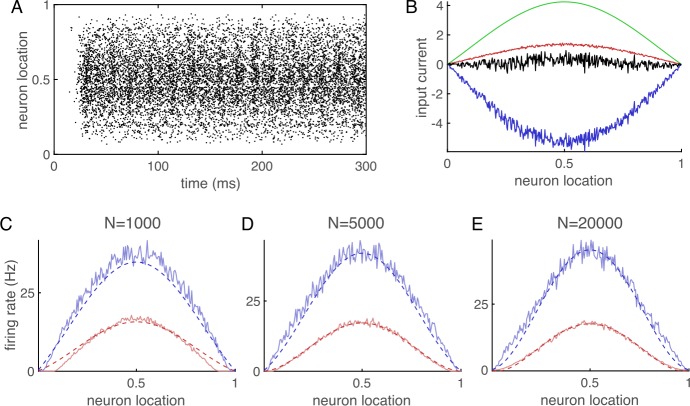
Example of an imbalanced network. (**A**)–(**E**) Same as Fig. 1 except external input is given by Eq. (18) with $c=0.15$, dashed lines in (**C**)–(**E**) are given by summing Eq. (13) numerically up to $m=20$, and dotted lines are not shown because there does not exist a solution to Eq. (9)

Despite the break in balance as $N\to \infty $, network simulations still show asynchronous-irregular spiking activity (Fig. 3(A)) and approximate excitatory-inhibitory balance (Fig. 3(B)) at finite *N*. Comparing Eq. (13) to firing rates from simulations shows that it is still somewhat accurate for multiple values of *N* (Fig. 3(C)–(E)).

Interestingly, the break in balance is not highly apparent in Fig. 3(B), as the currents still appear to be approximately balanced. Our mathematical analysis shows that the mean total input current cannot remain $\mathcal{O}(1)$ for all *x* as $N\to \infty $. However, we expect the divergence to grow like $\mathcal{O}(\sqrt{N})$ when balance is broken [12], so very large values of *N* could be necessary for imbalance to become visible.

## Discussion

We extended the mean-field theory of firing rates in balanced networks to account for spatial connectivity structures in which connection probabilities depend on the spatial location of pre- and postsynaptic neurons without the translation invariance assumed in previous work. Any such network cannot maintain balance as $N\to \infty $ for every external input profile, and we derived conditions on the external input profile and connection probabilities required for balance to be possible. We also derived a finite *N* approximation to the firing rates that is applicable even when strict balance cannot be achieved as $N\to \infty $. We compared our theoretical results to large simulations of randomly connected integrate-and-fire neuron models. While the equations left some error at finite *N*, they captured the overall shape of firing rates at large values of *N*.

For balance to be realized, the firing rate profiles given by Eq. (12) need to be positive at all values of $x\in \varOmega $. While we did not derive explicit conditions on this positivity, the solution in Eq. (12) can be checked for positivity and should only be interpreted as a valid solution when rates are positive. When Eq. (12) gives negative rates for some $x\in \varOmega $, this would lead to mean firing approaching zero as $N\to \infty $ over some spatial locations. It is possible that the remaining neurons in the network that have nonzero firing rates could realize balance separately, forming a balanced sub-network. This possibility, which requires a nonlinear analysis, will be explored in future work.

We parameterized feedforward input as a time-constant, deterministic function ${\boldsymbol{{F}}}(x)$. In reality, cortical populations receive noisy, time-varying feedforward input from neurons in other cortical layers, cortical areas, and thalamus. These can be modeled more realistically by generating the spike trains of these presynaptic populations and assigning feedforward connectivity analogous to the recurrent connectivity considered here [15, 17, 22]. In the mean-field theory, this gives ${\boldsymbol{F}}(x)=[\mathcal{W}_{F} r_{F}](x)$ where $r_{F}(y)$ quantifies the spatial profile of firing rates in the presynaptic population, which might live on a different space $y\in \varOmega _{y}$, and $\mathcal{W}_{F}$ is the mean-field connectivity kernel defined analogously to $\mathcal{W}$. In this case, Eq. (9) admits a solution, *i.e.*, balance can be achieved, whenever the range of $\mathcal{W}_{F}$ is contained within the range of $\mathcal{W}$. See [17] for a development of this idea for networks with translationally invariance connectivity profiles. This provides a way to test the spatial structure of multi-layered cortical circuits for the ability to maintain balance.

In addition, balance requires that the fixed point realized by Eq. (12) is stable. Stability of firing rate fixed points in networks of integrate-and-fire neurons can be very complicated and is outside the scope of this study. Stability for integro-differential equations of the form $\dot{{\boldsymbol{r}}}=-{\boldsymbol{r}}+g(\mathcal{W}+{\boldsymbol{X}})$ can be analyzed more easily, especially when *g* is assumed to be linear at the fixed point. This approach to stability analysis can provide a heuristic approximation to stability in spiking networks, but instabilities can arise in spiking networks that are not captured by the integro-differential equation due to the inherent resonance of spiking neurons [29]. An in-depth analysis of stability and the dynamics that arise when the networks are destabilized would be an interesting direction for future studies.

We assumed that connectivity kernels are symmetric $w_{ab}(x,y)=w_{ab}(y,x)$ and that all four kernels share the same eigenfunctions. Extensions to this theory for asymmetric kernels and distinct eigenfunctions could be achieved using more general numerical or analytical approaches to solving integral equations that rely on singular value decompositions instead of eigenfunction decompositions (the two coincide under our assumptions) [21, 30, 31]. Such an extension would, for example, allow the analysis of visual cortical network models with pinwheel-shaped orientation hypercolumns. This could potentially be achieved analytically using methods to recast those networks in spherical coordinates [32] or numerically using real imaging of hypercolumn geometry and neural connectivity [18]. This could lead to studies that numerically or analytically probe which visual stimuli break excitatory-inhibitory balance. Since imbalanced networks generally lead to an amplification of firing rate responses [17], this would shed light on which visual stimuli patterns should be more salient under a balance network formalism.

In summary, our study extends the theory of balanced networks to more intricate spatial topologies, which opens the door to a number of additional lines of inquiry that could provide additional insights into the operation of cortical circuits.
